# MicroRNA-155-5p Targets JADE-1, Promoting Proliferation, Migration, and Invasion in Clear Cell Renal Cell Carcinoma Cells

**DOI:** 10.3390/ijms24097825

**Published:** 2023-04-25

**Authors:** Thomas Kalantzakos, Kailey Hooper, Sanjna Das, Travis Sullivan, David Canes, Alireza Moinzadeh, Kimberly Rieger-Christ

**Affiliations:** 1 Department of Translational Research, Lahey Hospital and Medical Center, Burlington, MA 01805, USA; 2 Department of Urology, Lahey Hospital and Medical Center, Burlington, MA 01805, USA

**Keywords:** miR-155-5p, Jade-1, Clear cell renal cell carcinoma (ccRCC), metastasis, Von-Hippel Lindau (VHL), microRNAs

## Abstract

Clear cell renal cell carcinoma (ccRCC) incidence has been rising in recent years, with strong association between differential microRNA (miRNA) expression and neoplastic progression. Specifically, overexpression of miR-155-5p has been associated with promoting aggressive cancer in ccRCC and other cancers. In this study, we further investigate the role of this miRNA and one of its protein targets, Jade-1, to better understand the mechanism behind aggressive forms of ccRCC. Jade-1, a tumor suppressor, is stabilized by Von-Hippel Lindau (VHL), which is frequently mutated in ccRCC. Experiments featuring downregulation of miR-155-5p in two ccRCC cell lines (786-O and Caki-1) attenuated their oncogenic potential and led to increased levels of Jade-1. Conversely, knockdown experiments with an anti-Jade-1 shRNA in 786-O and Caki-1 cells showed increased metastatic potential through elevated proliferation, migration, and invasion rates. In a mouse xenograft model, downregulation of miR-155 decreased the rate of tumor implantation and proliferation. Direct interaction between miR-155-5p and Jade-1 was confirmed through a 3′UTR luciferase reporter assay. These findings further elucidate the mechanism of action of miR-155-5p in driving an aggressive phenotype in ccRCC through its role in regulating Jade-1.

## 1. Introduction

Renal cell carcinoma (RCC) is a malignant neoplasm of the kidney that currently constitutes about 2% of all global cancer diagnoses [1]. RCC is heterogeneous and can be further partitioned histologically, with clear cell renal cell carcinoma (ccRCC) being the most common [2] and associated with the worst survival prognosis [1]. Incidence rates of these tumors have been steadily rising over the past two decades due to advanced cross-sectional imaging, with the greatest absolute increase in small renal masses (<4 cm) [3]. Despite the traditional association with good prognosis for these lesions [4] and the opportunity for early intervention conferred through advancement in diagnostics, approximately 20–30% of small renal masses are potentially aggressive lesions [5]. As a result, while pathologic stage has been the principal indicator of ccRCC progression [6], recent studies have focused on identifying alternative methods to classify and address these small renal masses. Biomarkers, which are molecular markers that can aid in predicting disease progression [7], can be used in this manner. The expression levels of protein [7], microRNAs (miRNAs) [8], long non-coding RNAs [9], and transcription factors [10] have shown utility in predicting patient outcomes in ccRCC patients.

MiRNAs are small, non-coding RNAs of about 22 nucleotides in length that generally function to repress mRNA expression by binding to the 3′UTR and inducing degradation [11]. Each miRNA can modulate the expression of numerous targets, and each transcript may be regulated by hundreds of miRNAs, creating a network of interaction between these factors and their downstream effectors [12]. Several algorithms have been created to help identify these potential targets by estimating potential base pair alignments of miRNAs and the UTRs of protein coding genes. While the human genome likely encodes over 2500 miRNAs (https://www.mirbase.org/, accessed on 23 January 2023), this study furthers the investigation of miR-155-5p, which has been shown to play a vital role in oncogenesis [13,14].

MiR-155-5p has been shown to act as a proinflammatory, oncogenic miRNA in a variety of cancers [15]. Specifically, upregulation of miR-155-5p promotes proliferation and metastasis by targeting various tumor suppressing genes in bladder [16], colorectal [17,18], and breast cancer [19]. In addition, increased levels of miR-155-5p have been associated with poorer survival in several cancers, including pancreatic [20], bladder [21], hepatocellular [22], and non-small cell lung cancer [23]. Previous studies also indicate that miR-155-5p plays a role in ccRCC development and progression [24,25]. In individuals diagnosed with RCC, miR-155-5p upregulation was associated with decreased time to progression [24,26]. Additionally, miR-155-5p induces epithelial–mesenchymal transition (EMT) in RCC and therefore promotes cellular processes associated with metastasis [13]. The oncogenic effects of miR-155-5p have been demonstrated at both the organismal and cellular level; in a xenograft mouse model, miR-155-5p promoted growth of RCC [13] neoplasms, and in RCC cells, transfection with anti-miR-155-5p suppressed growth while miR-155-5p mimic promoted proliferation and migratory ability [13]. Moreover, our previous investigation noted that increased levels of miR-155-5p were associated with pT1 small renal masses that later progressed to metastatic disease [27]. The impact of a specific miRNA on cell behavior is through the transcripts and pathways with which they interact, allowing miRNA to regulate the entire network. As such, identifying protein targets is imperative to unraveling the mechanisms of oncogenesis.

Function of the Von Hippel–Lindau (VHL) protein is diminished in more than 80% of ccRCC cases, through allele deletion, promoter methylation, or mutations [28].The VHL protein normally functions in controlling cellular functions such as cell growth and division [29]. In ccRCC, VHL downregulation is mirrored by downregulation of Jade-1 [30]. The Jade-1 protein, a growth-suppressive ubiquitin ligase [31], is a suspected tumor suppressor thought to regulate apoptosis and cancer cell growth [32,33,34] and is predominantly localized in cell nuclei [35]. Two forms of Jade-1 have been reported: a full-length protein Jade-1L (95 kD) and a smaller splice variant Jade-1S (58 kD), the smaller form being the one most well described [36]. Jade-1 has previously been noted for its strong interaction with VHL [29], and it is suspected that VHL stabilizes and prolongs Jade-1 half-life [29]. Furthermore, in cell lines without VHL, Jade-1 degradation was significantly increased [33]. Clinically, Jade-1 expression has been associated with RCC irrespective of VHL [33], together suggesting an important role for Jade-1 in ccRCC development, and RCC patients with lower levels of Jade-1 had significantly shorter survival [34].

Here, miR-155-5p and Jade-1 are investigated in two ccRCC cell lines; in particular, through their connection to the key metastatic processes of proliferation, migration, and invasion. In addition, this study seeks to clarify the potential role of the interaction between miR-155-5p and Jade-1 and how this may affect phenotypes associated with metastasis in ccRCC.

## 2. Results

### 2.1. Survival Analyses

**Figure 1:**
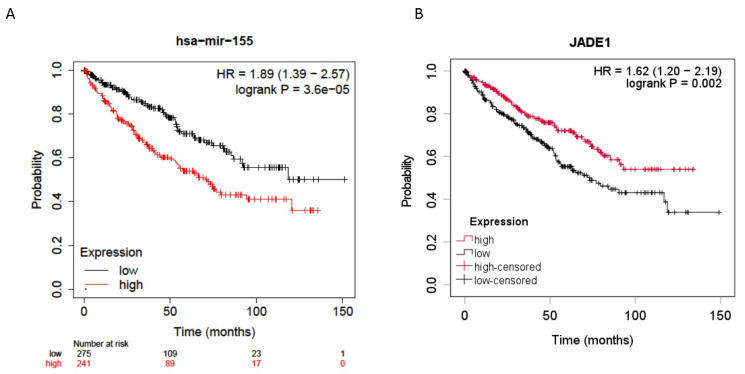
Overall survival data for all stage ccRCC patients from the TCGA dataset. (**A**) Higher miR-155-5p expression in ccRCC tumors was significantly associated with poorer overall survival. (**B**) Lower Jade-1 expression in ccRCC tumors was significantly associated with poorer overall survival.

Jade-1 was investigated because it was identified by four prediction algorithms to be a likely target of miR-155-5p, and its dysregulation has been implicated in ccRCC oncogenesis [33,34]. Expression levels of mir-155-5p (*n* = 516) and Jade-1 (*n* = 528) from the ccRCC TCGA dataset [37] were examined for their association with overall survival. All stages of ccRCC were included, and the median expression level was used as the cutoff. Higher expression levels of miR-155-5p and lower levels of Jade-1 transcript expression were associated with significantly poorer overall survival (HR = 1.89, 95% CI 1.39–2.57 *p* < 0.001 and HR = 1.62, 95% CI 1.20–2.19 *p* = 0.002, respectively) (Figure 1).

### 2.2. Knockdown of miR-155-5p and Jade-1

**Figure 2:**
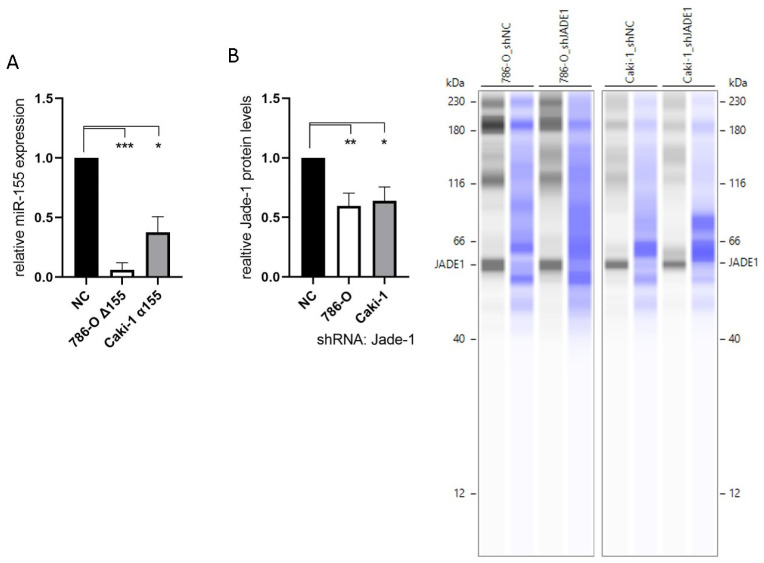
Knockdown of miR-155-5p and Jade-1. (**A**) Expression levels of miR-155-5p following CRISPR/Cas9 knockout in cell line 786-O (786-O Δ155) and lentiviral transduction with anti-miR-155-5p in Caki-1 (Caki-1 α155). (**B**) Protein levels of Jade-1 following lentiviral shRNA transduction of 786-O and Caki-1. Representative Simple Western™ images inset to the right: the first lane of each pair corresponds to JADE-1 and the second is total protein detected for the same lane. * *p* < 0.05, ** *p* < 0.01, *** *p* < 0.001.

To investigate the effects of miR-155-5p and Jade-1 in ccRCC cell lines, stable knockdown lines were established. The transcript levels of miR-155-5p were attenuated through CRISPR/Cas9 knockout in cell line 786-O (Figure 1A). PCR assaying and two independent rounds of Sanger sequencing were utilized to ensure deletion of the miR-155-5p genomic region (see Supplementary Materials for details). Likewise, lentiviral transduction of anti-miR-155-5p in cell line Caki-1 led to diminished expression of miR-155-5p (Figure 2A). Additionally, Jade-1 protein levels were significantly reduced following transfection with lentiviral shRNA constructs with unique sequences in 786-O and Caki-1 (Figure 2B). Western blot analysis resulted in a consistent band across all samples and gels of ~60 kD, which is consistent in size with the Jade-1S splice variant. We did not observe consistent bands in the molecular weight range of Jade-1L.

### 2.3. miR-155-5p Directly Targets the 3′UTR of Jade-1

**Figure 3:**
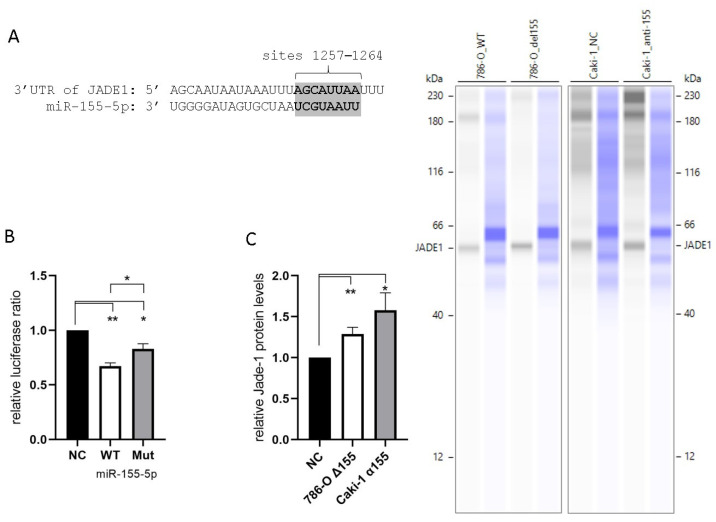
MiR-155-5p targets the 3′UTR of Jade-1. (**A**) The consensus binding site for miR-155-5p at positions 1257–1264 of the Jade-1 3′UTR. (**B**) Luciferase activity with the WT Jade-1 3′UTR and a mutant site compared to scramble negative control. (**C**) Protein expression of Jade-1 significantly increased following knockdown of miR-155-5p in 786-O and Caki-1 cell lines. Representative Simple Western™ images inset to the right: the first lane of each pair corresponds to JADE-1 and the second is total protein detected for the same lane. * *p* < 0.05, ** *p* < 0.01.

All four of the prediction algorithms we utilized identified Jade-1 as a target of miR-155-5p. Each algorithm identified a site in the 3′UTR at positions 1257–1264 (Figure 3A). In addition, two algorithms identified a site at positions 492–498, and a single algorithm identified a third site at positions 77–90. Unique plasmids were prepared containing the WT Jade-1 3′UTR as well as a 3′UTR with a mutated 1257–1264 site (see supplemental materials for details). A luciferase reporter assay was performed to confirm the interaction of miR-155-5p and the Jade-1 3′UTR. A significant reduction in the Renilla/Firefly luminescence ratio was observed 24 h after co-transfection of miR-155-5p with either plasmid, compared to negative control in 786-O cells (Figure 3B). In addition, there was a significant reduction in the luminescence ratio of the WT in comparison to the mutant plasmid. These results indicate that miR-155-5p directly binds to the 3′UTR of Jade-1. Furthermore, the protein expression levels of Jade-1 were significantly increased when miR-155-5p was knocked down in both 786-O and Caki-1 cells (Figure 3C).

### 2.4. miR-155-5p and Jade-1 Affect Phenotypes Associated with Metastasis in ccRCC Cell Lines

**Figure 4:**
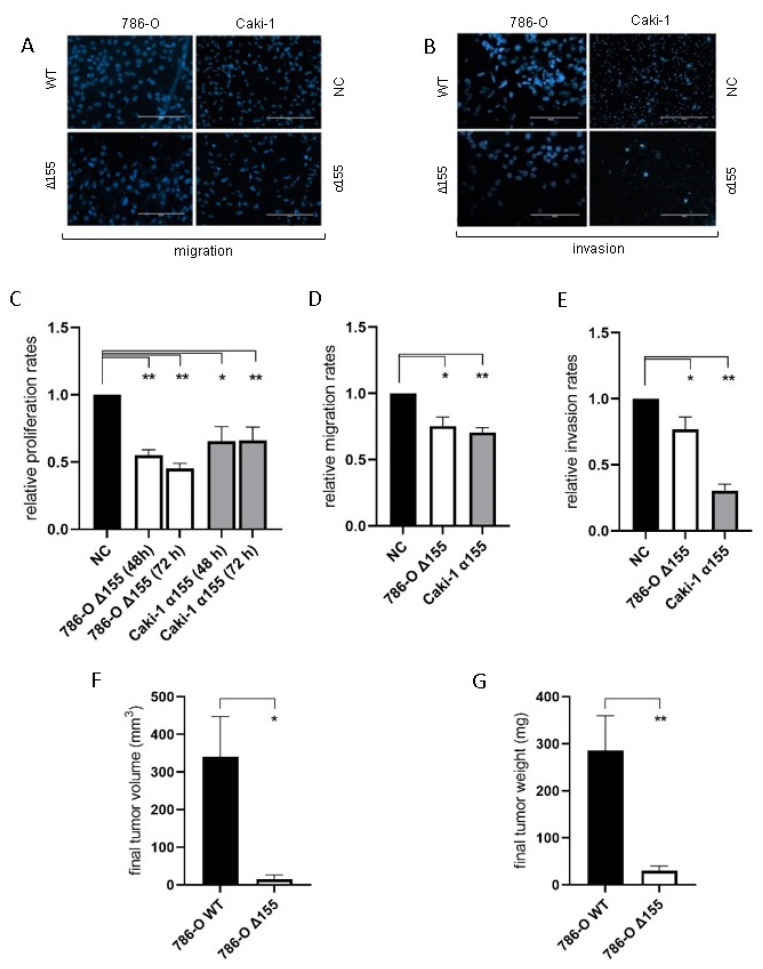
The reduction in levels of miR-155-5p significantly impacted cell attributes associated with metastasis in both ccRCC cell lines. (**A,B**) Representative images of the migration and invasion assays (scale bars represent 200µm). With decreased levels of miR-155-5p decreases for in vitro (**C**) proliferation, (**D**) migration, and (**E**) invasion rates were observed. In vivo xenograft tumor results for (**F**) tumor volume and (**G**) tumor weight. * *p* < 0.05, ** *p* < 0.01.

Reduction of miR-155-5p expression significantly decreased the rates of proliferation, migration, and invasion for both 786-O and Caki-1 cell lines in vitro compared to negative controls (Figure 4A–E). In addition, the rate of tumor implantation decreased significantly for the miR-155 knockdown line in vivo (20% for Δ155 cells vs. 90% for WT, *p* = 0.006), and the reduction in miR-155 expression significantly diminished ccRCC tumor growth in vivo (Figure 4F,G).

**Figure 5:**
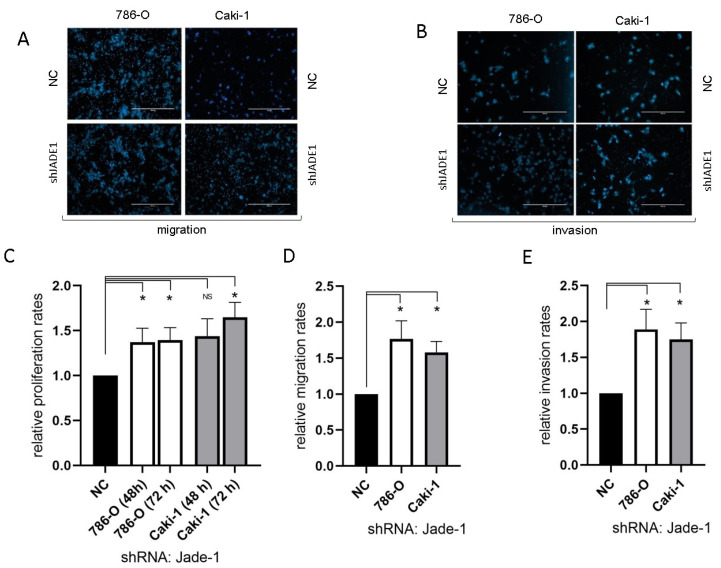
The reduction in the level of Jade-1 significantly enhanced cell attributes associated with the metastatic phenotype in both ccRCC cell lines. (**A**,**B**) Representative images of the migration and invasion assays (scale bars represent 200µm). (**C**) Cell proliferation following transfection with anti-Jade-1 shRNA. (**D**) Cell migration following transfection with anti-Jade-1 shRNA. (**E**) Cell invasion following transfection with anti-Jade-1 shRNA * *p* < 0.05.

Reduced levels of Jade-1 significantly increased the rates of proliferation, migration, and invasion for both 786-O and Caki-1 cell lines compared to negative controls (Figure 5).

## 3. Discussion

MiRNAs are important regulators of gene expression and have been implicated in cancer, both as oncogenic and tumor suppressive players [38]. This is especially true for ccRCC, where miRNAs such as miR-15a [39], miR-21 [40], miR-221 [41] and miR-149-5p [42] have been identified in connection with the disease. In addition, miRNAs have the potential to serve as biomarkers and may help to serve in the management of RCC [43]. Of particular interest is miR-155-5p, which has been explored both in terms of its role in promoting EMT in ccRCC [13,44,45] and its link with poorer patient outcomes [27,46,47]. For clinicians, identifying and addressing recurrence is vital in managing disease, and miR-155-5p specifically has been linked to increased recurrence risk [38]. Within ccRCC specifically, the oncogenic characteristics of miR-155-5p have been confirmed in previous work by our lab [27], which identified an association between high expression of miR-155-5p in stage I ccRCC and progression to metastatic disease. MiR-155-5p has also been demonstrated to mediate characteristics of ccRCC progression such as proliferation, migration, and invasion [13,44,45]. Furthermore, other studies have examined the utility of miR-155-5p as a biomarker for prognostic outcomes in those with ccRCC [48,49]. Beyond prognosis, a recent paper by Sequeira et al. 2022 found that miR-155-5p aided in accurate detection of RCC from patient plasma [50].

The evidence detailed in this study further supports the oncogenic role of miR-155-5p in ccRCC. Expression levels of miR-155-5p in the ccRCC TCGA dataset demonstrate a significant association between higher levels of miR-155-5p and poorer overall survival (Figure 1A). We established stable lines knocking down miR-155-5p in Caki-1 cells with an anti-miR, and in 786-O cells via CRISPR/Cas9 (Figure 2A). Proliferation, migration, and invasion were significantly decreased in both cell lines following knockdown (Figure 4), supporting the role of miR-155-5p in driving ccRCC progression. In addition, in a mouse xenograft model, the knockdown of miR-155 expression significantly decreased the rate of tumor implantation and proliferation (Figure 4). Western blotting in both stable lines revealed an increase in Jade-1 compared to negative control, suggesting an association between these two factors. A limitation of this study is that we limited the in vivo study to measurements of tumor size and implantation rate, but we did not specifically monitor for metastasis, nor did we investigate other protein markers specifically related to invasiveness or the EMT phenotype.

Direct binding of miR-155-5p to the 3′UTR was confirmed with a luciferase assay (Figure 3B). Luciferase expression was significantly lower when transfected with the WT 3′UTR and miR-155-5p compared to WT and negative control (*p* < 0.01) or mutant and miR-155-5p (*p* < 0.05). Interestingly, even with mutation of the 1257–1267 binding site of the Jade-1 3′UTR, a significant reduction in luciferase ratio compared to negative control was observed (*p* < 0.05), but significantly less so than the WT plasmid. This significant reduction for the mutated plasmid suggests the presence of miR-155-5p binding not only at the 1257–1267 site but also to additional target site(s) within the Jade-1 3′UTR—possibly site 492–498 and/or site 77–90. A limitation of our work is that we only investigated a single region of the Jade-1 3′UTR.

Lower levels of Jade-1 transcript expression were associated with significantly poorer overall survival in the ccRCC TCGA dataset (Figure 1B), the converse of the association for miR-155-5p. Likewise for the TCGA dataset, lower levels of Jade-1 were associated with significantly poorer overall survival in lung adenocarcinoma and prostate cancer (https://www.proteinatlas.org/, accessed on 23 January 2023). We examined the effects of Jade-1 on the cancer phenotype in ccRCC cell lines by establishing stable cell lines with a lentiviral anti-Jade-1 shRNA (Figure 2B). Proliferation, migration, and invasion were increased in both cell lines compared to negative control following knockdown of Jade-1 (Figure 5). We note a limitation of this study: although it would have been interesting to try and restore the levels of Jade-1 in an effort to investigate if increased levels of Jade-1 are associated with a less aggressive phenotype, it has been demonstrated that artificially increasing levels of Jade-1 can be cytotoxic [36]; therefore, we limited our investigation to inferring the effects of Jade-1 based on knockdown of the protein.

Jade-1 expression is shown here to be at least partially regulated by miR-155-5p. The strengths of our study include that to our knowledge, this study is the first to investigate the regulation of Jade-1 by non-coding RNA in ccRCC. The significance of Jade-1 as a tumor suppressor has previously been identified, particularly through its association with VHL [29]. Jade-1 expression is stabilized by VHL [29], and in the absence of VHL, Jade-1 degradation was significantly increased [33]. Within ccRCC specifically, Jade-1 expression has been shown to be prognostic for renal cancer regardless of VHL expression [33]. This finding is supported by the present study, as Jade-1 knockdown enhanced the aggressiveness in the two cell lines we tested, which included both VHL mutant (786-O) and wild type (Caki-1) cell lines. In addition to its interaction with VHL, the tumor-suppressive role of Jade-1 has also been demonstrated through its regulation as a transcription factor of AKT [51] and beta-catenin [52].

To our knowledge, there are no prior reports of Jade-1 with respect to the metastatic phenotypes of proliferation, migration, and invasion in ccRCC, though there are studies highlighting the role of VHL in this regard [53,54,55]. Due to the prominent role of VHL in ccRCC, and its reported strong interaction with Jade-1, this is a potential mechanistic axis for study. In pancreatic cancer stem cells, Jade-1 has been implicated in the EMT phenotype through the AKT/mTOR pathway [56]. Additionally, Zeng et al. [51] reported that reduced Jade-1 expression is a poor prognostic factor in ccRCC and is associated with activation of an AKT signature. In light of these details, and the well supported role of AKT/mTOR in regard to EMT in ccRCC [57], increased scrutiny of Jade-1 as a tumor suppressor of ccRCC and a potential agent inhibiting the EMT phenotype is much needed.

## 4. Materials and Methods

### 4.1. Cell Culture

The human ccRCC lines 786-O (ATCC^®^ CRL-1932™) and Caki-1 (ATCC^®^ HTB-46™) (American Type Culture Collection, Manassas, VA, USA) were cultured under standard conditions (37 °C, 5% CO_2_). 786-O, a VHL mutant RCC cell line with altered HIF and VEGF pathways, was derived from a primary epithelial clear cell adenocarcinoma and maintained in RPMI 1640 (ATCC). Caki-1, derived from a metastatic site on the skin, is a VHL wild type RCC cell line characterized by high VEGF production, and was grown in McCoy’s 5A media (ATCC). All media were supplemented with 10% fetal bovine serum, penicillin/streptomycin, and L-glutamine.

### 4.2. Identification of miR-155-5p Protein Targets

Potential protein targets of miR-155-5p were identified using four prediction algorithms: TargetScan v7.0 (https://www.targetscan.org/, accessed on 11 October 2021) [58], miRmap (https://mirmap.ezlab.org/, accessed on 11 October 2021) [59], miRDB (https://mirdb.org/, accessed on 11 October 2021) [60], and microT-CDS v5.0 (https://diana.e-ce.uth.gr/home, accessed on 11 October 2021) [61]. We screened for protein targets that were predicted by all of these algorithms. From a consensus list, the potential protein targets of miR-155-5p were screened through an extensive literature search focusing on reported tumor suppressor genes identified as being dysregulated in ccRCC. Subsequently, we finalized candidates using the Human Protein Atlas (https://www.proteinatlas.org/, accessed 18 October 2021) [62]. We focused on downregulated tumor suppressor genes associated with poor outcomes in both early- and later-stage ccRCC, using the TCGA dataset.

### 4.3. Knockdown of miR-155 by CRISPR/Cas9

Parental wild type (WT) 786-O cells were used to generate a miR-155 knockout (Δ155) cell line. Knockout cells were generated by Synthego (Redwood City, CA, USA) using single guide RNAs (sgRNA) specific to miR-155. The two sgRNA sequences used were CAGGUGGCACAAACCAGGAA and AUGGAACAAAUUGCUGCCGU. sgRNAs and SpCas9 were transfected into cells by optimized electroporation to form a ribonucleoprotein (RNP) complex. Successful excision was verified by PCR followed by Sanger sequencing, using the forward TGCCTAAAGGTAACAATGTCATCT and reverse TGAACAAGCCAAAACCTGCA PCR primers. Δ155 cells were enriched through four rounds of limiting dilutions. For each repeat, DNA was isolated from cells using the Qiagen QIAamp DNA Blood Mini Kit (51104, Qiagen, Hilden, Germany). Subsequently, PCR was conducted using a second set of custom primers (forward TCACTCCAGCTTTATAACCGCA and reverse GGTCACTGGGATGTTCAACCT), also designed to flank the miR-155 region, and deletion was verified by agarose gel electrophoresis. Additionally, the deletion of miR-155 was confirmed in the selected line via sequencing by a third party (Azenta, Burlington, MA, USA).

### 4.4. Lentiviral Transduction of ccRCC Cells with shRNA and Anti-miR

Lentiviral shRNA constructs targeting Jade-1 were obtained from Origene (Rockville, MD, USA), while anti miR-155-5p miArrest™ construct was acquired from Genecopoeia (Rockville, MD, USA). The individual manufacturer’s protocols were used to establish stable cell lines for each construct in addition to negative control. Caki-1 and 786-O WT cells were transduced with the Jade-1 shRNA construct, with unique sequences used for each cell line (Caki-1: TL302510VA-GTTGGAGGATGAGTTCTACACCTTCGTCA and 786-O: TL302510VB-GACGGCAATGAGATGGTGTTCTGTGACAA) as well as a corresponding scrambled negative control. Caki-1 cells exclusively were transduced with the anti-miR-155-5p construct and corresponding scrambled negative control. Stable parental cell strains of shRNA constructs targeting Jade-1 and anti-miR-155-5p (α-miR-155-5p) were maintained in media supplemented with selection agent (puromycin and hygromycin respectively) while cell assays were performed with daughter cells in media devoid of selection agent.

### 4.5. Real-Time PCR of miR-155-5p Expression

Relative expression levels of miR-155-5p were measured in the Δ155 and α-miR-155-5p cell lines and compared with controls via Real-Time PCR. Reverse transcription of RNA and expression measurements of miR-155-5p and the normalization control RNU43 were assessed using the TaqMan system (assay IDs 467534 and 001095, Applied Biosystems, Foster City, CA, USA) following the manufacturer’s instructions on a CFX thermal cycler (Bio-Rad, Hercules, CA, USA). The normalization of miR-155-5p expression was performed using the comparative C(T) method [63].

### 4.6. Cell Proliferation Assay

Cells were seeded into 35 mm dishes (Corning Costar Corporation, Cambridge, MA, USA) in duplicate at a density of 2 × 10^4^ cells/mL, determined using a hemocytometer (American Optical Corporation, Buffalo, NY, USA). Forty-eight [48] hours after culturing, RealTime-Glo™ MT Cell Viability Assay (Promega, Madison, WI, USA) reagents were added to each dish at a 1:2000 dilution. Luminescence readings were obtained utilizing the GloMax^®^ 20/20 Luminometer (Promega, Madison, WI, USA) 1 h after the addition of the Cell Viability reagents. This process was repeated at 72 h after culturing.

### 4.7. Cell Migration and Invasion Assays

In vitro migration and invasion assays were conducted using modified Boyden chambers containing Transwell membrane filter inserts (8 μm pores, Corning) in 24 well plate reservoirs. Migration assays were conducted by planting chambers in wells filled with serum free medium supplemented with fibronectin (10 μg/mL). For the invasion assay for the wild type and Δ155 786-O cells, was run with the upper surface of the membrane coated with Matrigel (Becton-Dickinson, Franklin Lakes, NJ, USA), diluted 1:80 and placed in wells filled with 10% media. For the remaining 786-O and Caki-1 cell lines, Corning^®^ BioCoat™ Matrigel^®^ Invasion Chamber, 8.0 µm PET Membrane 24-well permeable supports (354480, Corning Costar Corporation, Cambridge, MA, USA) were used. For all invasion assays, cells were seeded in serum-free media on the upper surface of the membrane and allowed to move towards the underside of the membrane for 24 h (786-O) or 36 h (Caki-1) cell lines, at 37 °C. For each assay, cells that crossed the membrane were fixed in 10% *w*/*v* neutral-buffered formalin (Simport, QC, Canada) and stained with DAPI (Invitrogen, Carlsbad, CA, USA) (1:500 dilution in PBS, 1% Triton X-100).

Cell counts for all migration and invasion assays of wild type and Δ155 786-O cells were obtained using a fluorescent capable microscope (Evos; Advanced Microscopy Group, Bothwell, WA, USA)—three unique image frames per well, and three wells per individual experiment. Cell counts for the remaining invasion assays of 786-O and Caki-1 cell lines were obtained from four unique image frames per well and two wells per individual experiment.

### 4.8. Xenograft Model

The effects of miR-155 on in vivo ccRCC cell growth were explored in a xenograft model using ten nude athymic mice (Jackson Laboratories, Bar Harbor, ME, USA). All mice were housed in a facility with a controlled humidity, room temperature, and light cycle. They were fed in the isolation cages with access to food and water ad libitum. 786-O WT (negative control) and 786-O CRISPR edited Δ155 cells were subcutaneously inoculated into opposing flanks of the mice with a 20 G needle with a quantity of 5 × 10^6^ cells, administered in 200 uL of sterile 1× PBS. The cell lines were tested for Mouse Parvovirus (MPV/MVM) by Charles River Laboratories (Wilmington, MA, USA) prior to inoculation. After ten weeks, the mice were euthanized, and, subsequently, the tumors were removed, measured, and weighed. The tumor volume was calculated using the following formula: length × width^2^ × 3.14/6. Animal experiments were approved by the Institutional Animal Care and Use Committee of Lahey Hospital and Medical Center.

### 4.9. Luciferase Assay

Vectors were prepared with the full-length human Jade-1 3′UTR linked to the Firefly luciferase gene as well as the Renilla luciferase gene for normalization (Genecopoeia, Rockville, MD, USA). One vector comprised the WT 3′UTR of Jade-1 that contained the theoretical interaction sites of miR-155-5p. An additional vector was created to assess a potential binding site of miR-155-5p through site-directed mutagenesis. 786-O WT cells were seeded at a density of 5 × 10^4^ cells/mL into CELLSTAR 24-well dishes. After 24 h, the media was replaced with Opti-MEM™ Reduced Serum Medium (31985062, Invitrogen). Cells were then transfected with the appropriate vector (0.2 ug) along with either pre-miR-155-5p or pre-miR-Precursor Negative Control #1 (30 nM each, Invitrogen, Cat# 4464066, assay ID MC28440 and AM17110), delivered with Endofectin (Genecopoeia). Twenty-four hours after transfection, the cells were lysed following the manufacturer’s protocol, and luminescence was measured using the Luc-Pair™ Duo-Luciferase Assay kit (Genecopoeia) using a GloMax luminometer (Promega). Firefly luminescence was normalized relative to Renilla luminescence.

### 4.10. Western Blot Analysis

Cells were lysed in 200 μL/well of boiled 1× SDS-Laemmli (250 mM Tris-HCl, 4% SDS, 10% glycerol, 0.003% bromophenol blue) from dishes displaying 70–80% confluency. Lysates were obtained by scraping the dishes manually, followed by shearing with a 24-gauge needle. Protein concentration of each sample was determined via the BCA assay (Pierce, Waltham, MA, USA). Lysates were standardized for total protein concentration and volume using ProteinSimple sample buffer containing DTT, heated at 95 °C for 5 min, then loaded in a unique well of a 12–230 kDa separation module on the Simple Western Jess system (ProteinSimple, Santa Clara, CA, USA). Jade-1 primary antibody (PA5-30831, Invitrogen) at a concentration of 1:10 was diluted in Antibody Diluent 2 (ProteinSimple). Anti-rabbit secondary antibody (042-206, ProteinSimple) was used as supplied according to the manufacturer’s protocol. Normalization of Jade-1 values against total protein expression was conducted in a single capillary, using RePlex and Total Protein Detection reagents (ProteinSimple), and each sample was tested in duplicate.

### 4.11. Statistical Analysis

All experiments were performed with a minimum of three independent trials. For the cell assays, a two-tailed Welch’s *t*-test was conducted to determine if a statistically significant difference in traits exists for cells undergoing treatment compared to negative control. For the in vivo analyses of tumor volume and tumor weight, zero values (representing failed implantations) were excluded from the analyses. The corresponding plots depict the mean relative response rate for treated cells relative to negative controls (NC). Each trial utilized a negative control corresponding to the appropriate cell line, and the response values for each treatment are relative to the corresponding NC. Error bars represent the standard error of the mean for each treatment. Since NC values are set to one, no error bars are depicted for that condition. Although a unique NC was used for both cell lines, we display a single NC bar in each chart for purposes of clarity. The categorical analysis for tumor implantation was performed using Fisher’s Exact test.

The association of miR-155-5p with overall survival in ccRCC was investigated using the Pan-cancer Kaplan-Meier Plotter (http://kmplot.com/analysis/ accessed 27 September 2021) [64], which utilizes the TCGA miRNA expression dataset. In addition, the association of potential protein targets of miR-155-5p with overall survival were investigated for ccRCC, using the TCGA gene transcript expression dataset at the Human Protein Atlas. For both analyses, the median expression level was used as the cutoff, and groups were compared by the log-rank test. For all analyses, a *p*-value less than 0.05 was considered to be significant.

## 5. Conclusions

Upregulation of miR-155-5p has previously been established as an important risk factor of ccRCC progression and aggressiveness. In the present study, we demonstrate that this miRNA modulates proliferation, migration, and invasion in ccRCC cells, and that its upregulation contributes to the suppression of Jade-1 in ccRCC cells. Furthermore, we demonstrate that Jade-1 knockdown in ccRCC cells leads to increased proliferation, migration, and invasion. These results highlight the need for investigations of Jade-1 and miR-155-5p with respect to their treatment potential in the clinical setting.

## Data Availability

Data presented in this study are available on request from the corresponding author.

## Supplementary Materials

The supporting information can be downloaded at: https://www.mdpi.com/article/10.3390/ijms24097825/s1.

## References

[1] Padala S.A., Barsouk A., Thandra K.C., Saginala K., Mohammed A., Vakiti A., Rawla P., Barsouk A. (2020). Epidemiology of Renal Cell Carcinoma. World J. Oncol..

[2] Cairns P. (2011). Renal Cell Carcinoma. Cancer Biomark..

[3] Hollingsworth J.M., Miller D.C., Daignault S., Hollenbeck B.K. (2006). Rising incidence of small renal masses: A need to reassess treatment effect. J. Natl. Cancer Inst..

[4] Park S.-W., Lee S.S., Lee D.H., Kil Nam J., Chung M.K. (2017). Growth kinetics of small renal mass: Initial analysis of active surveillance registry. Investig. Clin. Urol..

[5] Wang Z.J., Westphalen A.C., Zagoria R.J. (2018). CT and MRI of small renal masses. Br. J. Radiol..

[6] Keegan K.A., Schupp C.W., Chamie K., Hellenthal N.J., Evans C.P., Koppie T.M. (2012). Histopathology of Surgically Treated Renal Cell Carcinoma: Survival Differences by Subtype and Stage. J. Urol..

[7] Crispen P.L., Boorjian S.A., Lohse C.M., Leibovich B.C., Kwon E.D. (2008). Predicting disease progression after nephrectomy for localized renal cell carcinoma: The utility of prognostic models and molecular biomarkers. Cancer.

[8] Gowrishankar B., Ibragimova I., Zhou Y., Slifker M.J., Devarajan K., Al-Saleem T., Uzzo R.G., Cairns P. (2013). MicroRNA expression signatures of stage, grade, and progression in clear cell RCC. Cancer Biol. Ther..

[9] Qu L., Wang Z.-L., Chen Q., Li Y.-M., He H.-W., Hsieh J.J., Xue S., Wu Z.-J., Liu B., Tang H. (2018). Prognostic Value of a Long Non-coding RNA Signature in Localized Clear Cell Renal Cell Carcinoma. Eur. Urol..

[10] Qin S., Shi X., Wang C., Jin P., Ma F. (2019). Transcription Factor and miRNA Interplays Can Manifest the Survival of ccRCC Patients. Cancers.

[11] O’Brien J., Hayder H., Zayed Y., Peng C. (2018). Overview of MicroRNA Biogenesis, Mechanisms of Actions, and Circulation. Front. Endocrinol..

[12] Ghafouri-Fard S., Shirvani-Farsani Z., Branicki W., Taheri M. (2020). MicroRNA Signature in Renal Cell Carcinoma. Front. Oncol..

[13] Lei Q.-Q., Huang Y., Li B., Han L., Lv C. (2021). MiR-155-5p promotes metastasis and epithelial–mesenchymal transition of renal cell carcinoma by targeting apoptosis-inducing factor. Int. J. Biol. Mark..

[14] Alivernini S., Gremese E., McSharry C., Tolusso B., Ferraccioli G., McInnes I.B., Kurowska-Stolarska M. (2018). MicroRNA-155—At the Critical Interface of Innate and Adaptive Immunity in Arthritis. Front. Immunol..

[15] Mahesh G., Biswas R. (2019). MicroRNA-155: A Master Regulator of Inflammation. J. Interf. Cytokine Res..

[16] Chen L., Yang X., Zhao J., Xiong M., Almaraihah R., Chen Z., Hou T. (2020). Circ_0008532 promotes bladder cancer progression by regulation of the miR-155-5p/miR-330-5p/MTGR1 axis. J. Exp. Clin. Cancer Res..

[17] Qu Y.L., Wang H.F., Sun Z.Q., Tang Y., Han X.N., Yu X.B., Liu K. (2015). Up-regulated miR-155-5p promotes cell proliferation, invasion and metastasis in colorectal carcinoma. Int. J. Clin. Exp. Pathol..

[18] Gao Y., Liu Z., Ding Z., Hou S., Li J., Jiang K. (2018). MicroRNA-155 increases colon cancer chemoresistance to cisplatin by targeting forkhead box O3. Oncol. Lett..

[19] Pasculli B., Barbano R., Fontana A., Biagini T., Di Viesti M.P., Rendina M., Valori V.M., Morritti M., Bravaccini S., Ravaioli S. (2020). Hsa-miR-155-5p Up-Regulation in Breast Cancer and Its Relevance for Treatment With Poly[ADP-Ribose] Polymerase 1 (PARP-1) Inhibitors. Front. Oncol..

[20] Greither T., Grochola L.F., Udelnow A., Lautenschläger C., Würl P., Taubert H. (2009). Elevated expression of microRNAs 155, 203, 210 and 222 in pancreatic tumors is associated with poorer survival. Int. J. Cancer.

[21] Wang H., Men C.P. (2015). Correlation of Increased Expression of MicroRNA-155 in Bladder Cancer and Prognosis. Lab. Med..

[22] Han Z.B., Chen H.Y., Fan J.W., Wu J.Y., Tang H.M., Peng Z.H. (2012). Up-regulation of microRNA-155 promotes cancer cell invasion and predicts poor survival of hepatocellular carcinoma following liver transplantation. J. Cancer Res. Clin. Oncol..

[23] Xu T.-P., Zhu C.-H., Zhang J., Xia R., Wu F.-L., Han L., Shen H., Liu L.-X., Shu Y.-Q. (2013). MicroRNA-155 expression has prognostic value in patients with non-small cell lung cancer and digestive system carcinomas. Asian Pac. J. Cancer Prev..

[24] Kajdasz A., Majer W., Kluzek K., Sobkowiak J., Milecki T., Derebecka N., Kwias Z., Bluyssen H.A.R., Wesoly J. (2021). Identification of RCC Subtype-Specific microRNAs–Meta-Analysis of High-Throughput RCC Tumor microRNA Expression Data. Cancers.

[25] Shiomi E., Kato R., Matsuura T., Maekawa S., Kato Y., Kanehira M., Takata R., Sugimure J., Ishida K., Abe T. (2021). Relationship between miR-155 expression and clear cell papillary renal cell carcinoma in the dialyzed kidney. IJU Case Rep..

[26] Merhautova J., Hezova R., Poprach A., Kovarikova A., Radova L., Svoboda M., Vyzula R., Demlova R., Slaby O. (2015). miR-155 and miR-484 Are Associated with Time to Progression in Metastatic Renal Cell Carcinoma Treated with Sunitinib. BioMed Res. Int..

[27] Moynihan M.J., Sullivan T.B., Burks E., Schober J., Calabrese M., Fredrick A., Kalantzakos T., Warrick J., Canes D., Raman J.D. (2020). MicroRNA profile in stage I clear cell renal cell carcinoma predicts progression to metastatic disease. Urol. Oncol. Semin. Orig. Investig..

[28] Razafinjatovo C., Bihr S., Mischo A., Vogl U., Schmidinger M., Moch H., Schraml P. (2016). Characterization of VHL missense mutations in sporadic clear cell renal cell carcinoma: Hotspots, affected binding domains, functional impact on pVHL and therapeutic relevance. BMC Cancer.

[29] Zhou M.I., Wang H., Ross J.J., Kuzmin I., Xu C., Cohen H.T. (2002). The von Hippel-Lindau Tumor Suppressor Stabilizes Novel Plant Homeodomain Protein Jade-1. J. Biol. Chem..

[30] Xiao-Fen W., Ting C., Jie L., Deng-Yang M., Qing-Feng Z., Xin L. (2016). Correlation analysis of VHL and Jade-1 gene expression in human renal cell carcinoma. Open Med..

[31] Foy R.L., Chitalia V.C., Panchenko M.V., Zeng L., Lopez D., Lee J.W., Rana S.V., Boletta A., Qian F., Tsiokas L. (2012). Polycystin-1 regulates the stability and ubiquitination of transcription factor Jade-1. Hum. Mol. Genet..

[32] Banumathy G., Cairns P. (2010). Signaling pathways in renal cell carcinoma. Cancer Biol. Ther..

[33] Hou M.I., Foy R.L., Chitalia V.C., Zhao J., Panchenko M.V., Wang H., Cohen H. (2005). Jade-1, a candidate renal tumor suppressor that promotes apoptosis. Proc. Natl. Acad. Sci. USA.

[34] Lian X., Duan X., Wu X., Li C., Chen S., Wang S., Cai Y., Weng Z. (2012). Expression and Clinical Significance of Von Hippel-Lindau Downstream Genes: Jade-1 and β-Catenin Related to Renal Cell Carcinoma. Urology.

[35] Panchenko M.V. (2016). Structure, function and regulation of Jade Family PHD Finger 1 (JADE1). Panchenko, M.V. Structure, function and regulation of jade family PHD finger 1 (JADE1). Gene.

[36] Siriwardana N.S., Meyer R.D., Panchenko M.V. (2015). The novel function of JADE1S in cytokinesis of epithelial cells. Cell Cycle.

[37] Ricketts C.J., De Cubas A.A., Fan H., Smith C.C., Lang M., Reznik E., Bowlby R., Gibb E.A., Akbani R., Beroukhim R. (2018). The Cancer Genome Atlas Comprehensive Molecular Characterization of Renal Cell Carcinoma. Cell Rep..

[38] Zhang B., Pan X., Cobb G.P., Anderson T.A. (2007). microRNAs as oncogenes and tumor suppressors. Dev. Biol..

[39] Kalantzakos T.J., Sullivan T.B., Sebel L.E., Canes D., Burks E.J., Moinzadeh A., Rieger-Christ K.M. (2021). MicroRNAs MiR-15a and MiR-26a cooperatively regulate O-GlcNAc-transferase to control proliferation in clear cell renal cell carcinoma. Cancer Biomark..

[40] Zaman M.S., Shahryari V., Deng G., Thamminana S., Saini S., Majid S., Chang I., Hirata H., Ueno K., Yamamura S. (2012). Up-regulation of microRNA-21 correlates with lower kidney cancer survival. PLoS ONE.

[41] Szabó Z., Szegedi K., Gombos K., Mahua C., Flaskó T., Harda K., Halmos G. (2016). Expression of miRNA-21 and miRNA-221 in clear cell renal cell carcinoma (ccRCC) and their possible role in the development of ccRCC. Urol. Oncol. Semin. Orig. Investig..

[42] Xie M., Lv Y., Liu Z., Zhang J., Liang C., Liao X., Liang R., Lin Y., Li Y. (2018). Identification and validation of a four-miRNA (miRNA-21-5p, miRNA-9-5p, miR-149-5p, and miRNA-30b-5p) prognosis signature in clear cell renal cell carcinoma. Cancer Manag. Res..

[43] Napolitano L., Orecchia L., Giulioni C., Carbonara U., Tavella G., Lizzio L., Fimognari D., De Palma A., Gheza A., Grosso A.-A. (2023). The Role of miRNA in the Management of Localized and Advanced Renal Masses, a Narrative Review of the Literature. Appl. Sci..

[44] Wu H., Wu H., Sun P., Zhu D., Ma M., Fan W. (2021). miR-155-5p Promotes Cell Proliferation and Migration of Clear Cell Renal Cell Carcinoma by Targeting PEG3. Urol. Int..

[45] Tao M., Zhou Y., Jin Y., Pu J. (2020). Blocking lncRNA MIR155HG/miR-155-5p/-3p inhibits proliferation, invasion and migration of clear cell renal cell carcinoma. Pathol. Res. Pract..

[46] Shinmei S., Sakamoto N., Goto K., Sentani K., Anami K., Hayashi T., Teishima J., Matsubara A., Oue N., Kitadai Y. (2013). MicroRNA-155 is a predictive marker for survival in patients with clear cell renal cell carcinoma. Int. J. Urol..

[47] Gao Y., Ma X., Yao Y., Li H., Fan Y., Zhang Y., Zhao C., Wang L., Ma M., Lei Z. (2016). miR-155 regulates the proliferation and invasion of clear cell renal cell carcinoma cells by targeting E2F2. Oncotarget.

[48] Zhou Q., Zhang Z.-Y., Ang X.-J., Hu C., Ouyang J. (2021). Construction of five microRNAs prognostic markers and a prognostic model for clear cell renal cell carcinoma. Transl. Cancer Res..

[49] Zhang J., Ye Y., Chang D.W., Lin S.-H., Huang M., Tannir N.M., Matin S., Karam J.A., Wood C.G., Chen Z.-N. (2018). Global and Targeted miRNA Expression Profiling in Clear Cell Renal Cell Carcinoma Tissues Potentially Links miR-155-5p and miR-210-3p to both Tumorigenesis and Recurrence. Am. J. Pathol..

[50] Sequeira J.P., Constâncio V., Salta S., Lobo J., Barros-Silva D., Carvalho-Maia C., Rodrigues J., Braga I., Henrique R., Jerónimo C. (2022). LiKidMiRs: A ddPCR-Based Panel of 4 Circulating miRNAs for Detection of Renal Cell Carcinoma. Cancers.

[51] Zeng L., Bai M., Mittal A.K., El-Jouni W., Zhou J., Cohen D.M., Zhou M.I., Cohen H.T. (2013). Candidate tumor suppressor and pVHL partner Jade-1 binds and inhibits AKT in renal cell carcinoma. Cancer Res..

[52] Chitalia V.C., Foy R.L., Bachschmid M.M., Zeng L., Panchenko M.V., Zhou M.I., Bharti A., Seldin D.C., Lecker S.H., Dominguez I. (2008). Jade-1 inhibits Wnt signalling by ubiquitylating beta-catenin and mediates Wnt pathway inhibition by pVHL. Nat. Cell. Biol..

[53] Ma X., Tan Z., Zhang Q., Ma K., Xiao J., Wang X., Wany Y., Zhong M., Wang Y., Li J. (2022). VHL Ser65 mutations enhance HIF2α signaling and promote epithelial-mesenchymal transition of renal cancer cells. Cell Biosci..

[54] Pantuck A.J., An J., Liu H., Rettig M.B. (2010). NF-kappaB-dependent plasticity of the epithelial to mesenchymal transition induced by Von Hippel-Lindau inactivation in renal cell carcinomas. Cancer Res..

[55] Zhu S., Ding W., Chen Y., Wang W., Xu R., Liu C., Liu X., Deng H. (2022). High VHL Expression Reverses Warburg Phenotype and Enhances Immunogenicity in Kidney Tumor Cells. Genom. Proteom. Bioinform..

[56] Zhou J., Wang H., Che J., Xu L., Yang W., Li Y., Zhou W. (2020). Silencing of microRNA-135b inhibits invasion, migration, and stemness of CD24+CD44+ pancreatic cancer stem cells through JADE-1-dependent AKT/mTOR pathway. Cancer Cell Int..

[57] Kumar A., Kumari N., Gupta V., Prasad R. (2018). Renal Cell Carcinoma: Molecular Aspects. Indian J. Clin. Biochem..

[58] Agarwal V., Bell G.W., Nam J.W., Bartel D.P. (2015). Predicting effective microRNA target sites in mammalian mRNAs. Elife.

[59] Vejnar C., Zdobnov E.M. (2012). MiRmap: Comprehensive prediction of microRNA target repression strength. Nucleic Acids Res..

[60] Chen Y., Wang X. (2019). miRDB: An online database for prediction of functional microRNA targets. Nucleic Acids Res..

[61] Paraskevopoulou M.D., Georgakilas G., Kostoulas N., Vlachos I.S., Vergoulis T., Reczko M., Filippidis C., Dalamagas T., Hatzigeorgiou A.G. (2013). DIANA-microT web server v5.0: Service integration into miRNA functional analysis workflows. Nucleic Acids Res..

[62] Uhlén M., Zhang C., Lee S., Sjöstedt E., Fagerberg L., Bidkhori G., Benfeitas R., Arif M., Liu Z., Edfors F. (2017). A pathology atlas of the human cancer transcriptome. Science.

[63] Keren A., Tzivoni D. (1991). Torsades de pointes: Prevention and therapy. Cardiovasc. Drugs Ther..

[64] Lánczky A., Győrffy B. (2021). Web-Based Survival Analysis Tool Tailored for Medical Research (KMplot): Development and Implementation. J. Med. Internet Res..

