# Supplementary material for: MicroRNA-155-5p Targets JADE-1, Promoting Proliferation, Migration, and Invasion in Clear Cell Renal Cell Carcinoma Cells
Source: Int J Mol Sci. 2023 Apr 25;24(9):7825. doi: 10.3390/ijms24097825 (PMC10178234; doi:10.3390/ijms24097825)

Verification of CRISPR/Cas9 genomic excision of miR-155

Synthego


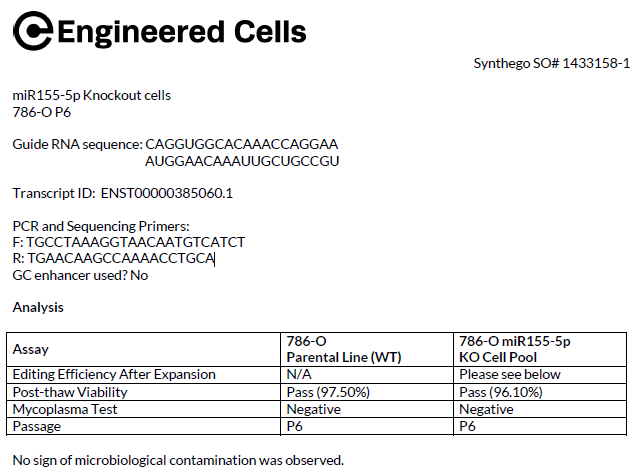


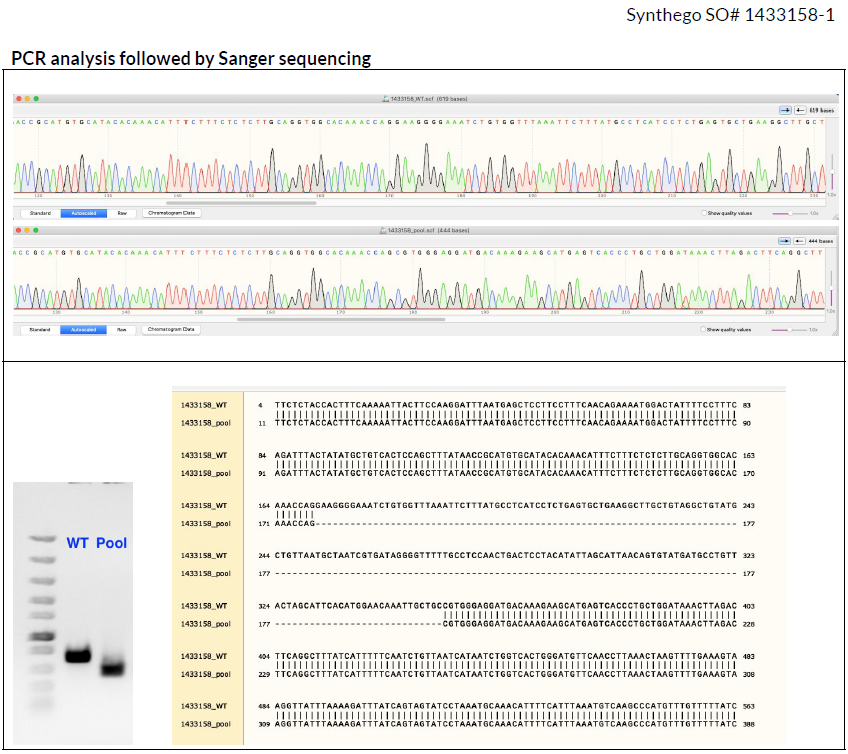


Third party (Azenta) confirmation of deletion of miR-155 via Sanger sequencing

Primers: forward TCACTCCAGCTTTATAACCGCA and reverse GGTCACTGGGATGTTCAACCT

WT

TGTGCATACACAAACATTTCTTTCTCTCTTGCAGGTGGCACAAACCAGGAAGGGGAAATCTGTGGTTTAAATTCTTTATGCCTCATCCTCTGAGTGCTGAAGGCTTGCTGTAGGCTGTATGCTGTTAATGCTAATCGTGATAGGGGTTTTTGCCTCCAACTGACTCCTACATATTAGCATTAACAGTGTATGATGCCTGTTACTAGCATTCACATGGAACAAATTGCTGCCGTGGGAGGATGACAAAGAAGCATGAGTCACCCTGCTGGATAAACTTAGACTTCAGGCTTTATCATTTTTCAATCTGTTAATCATAATCT

Del155

TGTGCATACACAAACATTTCTTTCTCTCTTGCAGGTGGCACAAACCAGCGTGGGAGGATGACAAAGAAGCATGAGTCACCCTGCTGGATAAACTTAGACTTCAGGCTTTATCATTTTTCAATCTGTTAATCATAATCT

Details for 3’UTR JADE-1 materials

JADE-1 (PHF17)

>hg38_knownGene_ENST00000226319.11 range=chr4:128831759-128875224 5'pad=0 3'pad=0 strand=+ repeatMasking=none

Start of WT 3’UTR for JADE-1

tgcaacagagatgatgcggaa

gccctttgggctcgtcattgggtttgctagaggagagctctgatgtgggg

gagaagcagaaacccattaatcctgagctacacaaacacatttacttgca

attcagattaatttttttccagagtcatttttaaatcatttttgtgagaa

gtttgtgttatttgcaacttgttgaggaaacagaagagtagattgtaacc

ataagacactgctaagactagaacccgaactgaacactaaaataaaaatg

aaatgttttaaagaaggcaaggcttaaataagcctcatgaatttttatag

ccctctgcattcttcccaaagcacaaactcatggttacctgaatataggg

aaccagatatggttcttgagaaaccctcatggtaccattcacagcccata

aagtttattttctaggactgtggtagatcttgaaatcatatttatatttg

gccctcaaggctatttttgttgcattatagcatataggcagcagctctga

agcttcagtaacactaagaaatttatgtgtaaatatagcagtcagggaag

agaattttaaaaaaggtcattattgaagaagctgaggggacagggtagag

ctgctgcaatatggagatttagggtaatatggcaaggtcctgctgcttga

agcctgtgagtgggttgtggatatgggactggtggagagtgagactgtta

ggaaagttgtatctatgaataagggcagttgaagtagaatttttaccagt

ccacttgaccttcttcttccctaaccactggctcttgagccagcttggga

tttccctggccattgccaatacctggccatctggcttccaatagtacagt

ggctactcaagttcaagcgaagaacttccagactctggtgactgttactt

cccagagccaaccactagtgcatatgttagggaatctgggcttccaacat

gaatggattccttaagaaaaaggaaaaaaaaaaaaaaaagaaaaaaagaa

aaaaaaaagaaaaaagaaaaaaagagaaaaaagcgaaataggttatattt

taaaaacaatagaaaggcaataagttgcgataagctcttactattgacca

aggttatacaggaaagagactgaagtgtacccttgaataggttttctgta

gtcagagttctaaactctaatttgtaacttggactttctaattgcaaatg

gcaataactattaagttatcagcaataataaatttagcattaaatttgag

tacaatgttttgtttttgcactccccatagtgcgtatgtattaagacagt

ggatagtgtttaggtcctgttaattttctttggaattcaatgtggttgtg

aatcaaacttaaggaaggaacgtttaaatagcaatgagatacagaattat

gggcctttggaacaagcccgacttcccctaaattctccttagtttgttaa

taccagtattcagattcctgattcatttatacatctgtttccatatggca

gggacattatgatacttaatgaataatgctttgaggagttctgcagttaa

ctttcaagtcttccagatgattgtcaacaacaaaaaaggcttattgaatc

ccatcttgctatgcaagttttatcagatgatcaaatagtagatctgatac

atccccattgtatgtacgacattttcaaaccaagtcttaacttttcaagg

acattttagtagctaattcaggggagggggaagaatgatgcaggttttta

gattgactgactatttttgagttatggggctcattttgaaagactgctgt

ccagatcagcttgttgctgcagataatagaaggttcttatgaatccaagt

tgtatattcacttgtaggataatttaaaaattagattttttttgcatatg

agcaaaaaccttttgctggatacaggagaaggttggactttatctacagt

tatcttttgattacagcaacagctctgggtgagagtagaatttatagagg

gataatttgtcaagccatagaaagaaaatctaaattaatctagtaagtgt

atgacctctcaccattttaagaggtatcagattcatttgcactattagga

atgctagttttgtgcaaaaataatgccttacctgttttttccccacattt

aggttgaaaagctttcaaatgttccaagttatgctgaaccaaaaaaaaca

aaacaaaaacaaaacaaaaaatattaaaaaaaacccacacaaaaacaaaa

cacacaaaaataaaaagcccacactttttattcctgcttcgaaatgcaaa

tggatagagcacggtttctctgacagtataatgatagctttgtgagttag

tttcatgtcatgctgggaactctctatgaggtggccataagcagcaacca

gcccaaacacccacttgcgttctattagtatggaaccatttgcatttgtt

ttttttaagctttatctttccttgtgcatcctgaccaagaaatatctttg

attatgattaatgtattatgtcaaaatgtaggctagttaaacttttgtaa

agttgcctggaatgtcatttgttaggttataaacacaagatctaaatgaa

gggttttatgtgttgtgtacaaatcttattttgaaatggacaaacttgtc

attacatttgtaaccttgtacagaggatttttcactatgtgcctagcttg

gtgtccattcagctaaaattgaaaaaaaaaaaaaggtgcatgaagagtta

aaaatcaaattaaagtatatgtagagatgactattttatattacatgacc

caatcctgtatttatttctaccccctttttgaaagtatttataaaactag

ttgaggacagctgtatttttttgttgaactatttagtagaattgtgcctt

tttgtctgtatgtgaataaatgctgtacattttgcaataca

Results of the four prediction algorithms used in this study

TargetScan


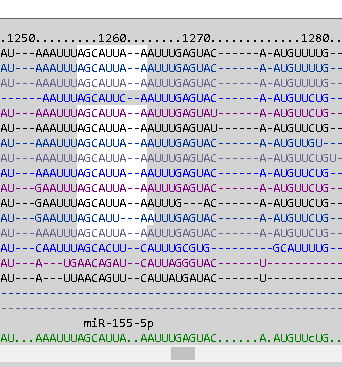


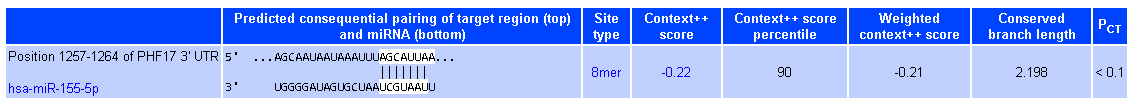


miRmapweb


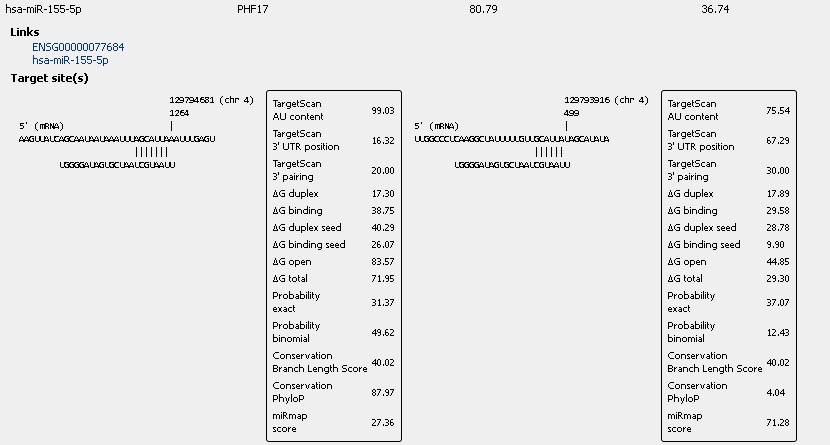


miRDB


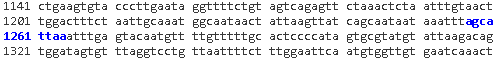


Diana Tool microT-CDS


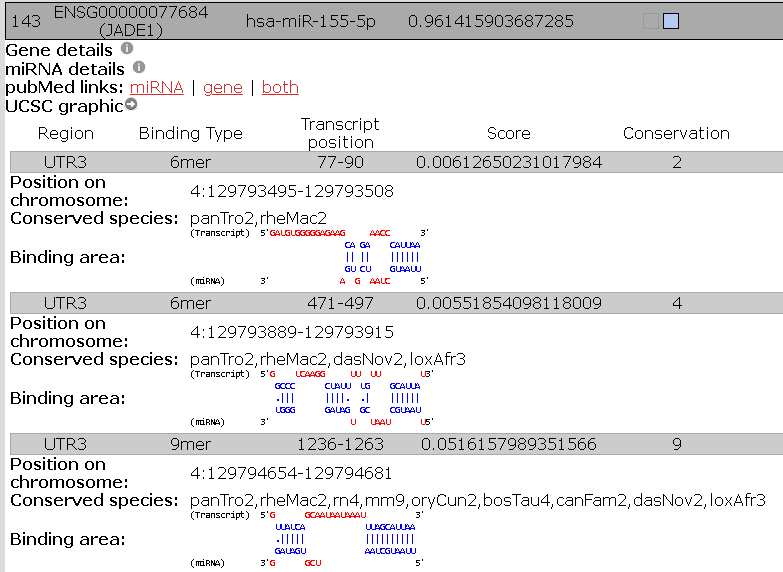


3’UTR mutant construct from Genecopeia

NM_199320.3

Mutation: AGCATTAA to TCGTAATT at nt 1257-1264

tgcaacagagatgatgcggaagccctttgggctcgtcattgggtttgctagaggagagctctgatgtgggggagaagcagaaacccattaatcctgagctacacaaacacatttacttgcaattcagattaatttttttccagagtcatttttaaatcatttttgtgagaagtttgtgttatttgcaacttgttgaggaaacagaagagtagattgtaaccataagacactgctaagactagaacccgaactgaacactaaaataaaaatgaaatgttttaaagaaggcaaggcttaaataagcctcatgaatttttatagccctctgcattcttcccaaagcacaaactcatggttacctgaatatagggaaccagatatggttcttgagaaaccctcatggtaccattcacagcccataaagtttattttctaggactgtggtagatcttgaaatcatatttatatttggccctcaaggctatttttgttgcattatagcatataggcagcagctctgaagcttcagtaacactaagaaatttatgtgtaaatatagcagtcagggaagagaattttaaaaaaggtcattattgaagaagctgaggggacagggtagagctgctgcaatatggagatttagggtaatatggcaaggtcctgctgcttgaagcctgtgagtgggttgtggatatgggactggtggagagtgagactgttaggaaagttgtatctatgaataagggcagttgaagtagaatttttaccagtccacttgaccttcttcttccctaaccactggctcttgagccagcttgggatttccctggccattgccaatacctggccatctggcttccaatagtacagtggctactcaagttcaagcgaagaacttccagactctggtgactgttacttcccagagccaaccactagtgcatatgttagggaatctgggcttccaacatgaatggattccttaagaaaaaggaaaaaaaaaaaaaaaagaaaaaaagaaaaaaaaaagaaaaaagaaaaaaagagaaaaaagcgaaataggttatattttaaaaacaatagaaaggcaataagttgcgataagctcttactattgaccaaggttatacaggaaagagactgaagtgtacccttgaataggttttctgtagtcagagttctaaactctaatttgtaacttggactttctaattgcaaatggcaataactattaagttatcagcaataataaatttTCGTAATTatttgagtacaatgttttgtttttgcactccccatagtgcgtatgtattaagacagtggatagtgtttaggtcctgttaattttctttggaattcaatgtggttgtgaatcaaacttaaggaaggaacgtttaaatagcaatgagatacagaattatgggcctttggaacaagcccgacttcccctaaattctccttagtttgttaataccagtattcagattcctgattcatttatacatctgtttccatatggcagggacattatgatacttaatgaataatgctttgaggagttctgcagttaactttcaagtcttccagatgattgtcaacaacaaaaaaggcttattgaatcccatcttgctatgcaagttttatcagatgatcaaatagtagatctgatacatccccattgtatgtacgacattttcaaaccaagtcttaacttttcaaggacattttagtagctaattcaggggagggggaagaatgatgcaggtttttagattgactgactatttttgagttatggggctcattttgaaagactgctgtccagatcagcttgttgctgcagataatagaaggttcttatgaatccaagttgtatattcacttgtaggataatttaaaaattagattttttttgcatatgagcaaaaaccttttgctggatacaggagaaggttggactttatctacagttatcttttgattacagcaacagctctgggtgagagtagaatttatagagggataatttgtcaagccatagaaagaaaatctaaattaatctagtaagtgtatgacctctcaccattttaagaggtatcagattcatttgcactattaggaatgctagttttgtgcaaaaataatgccttacctgttttttccccacatttaggttgaaaagctttcaaatgttccaagttatgctgaaccaaaaaaaacaaaacaaaaacaaaacaaaaaatattaaaaaaaacccacacaaaaacaaaacacacaaaaataaaaagcccacactttttattcctgcttcgaaatgcaaatggatagagcacggtttctctgacagtataatgatagctttgtgagttagtttcatgtcatgctgggaactctctatgaggtggccataagcagcaaccagcccaaacacccacttgcgttctattagtatggaaccatttgcatttgttttttttaagctttatctttccttgtgcatcctgaccaagaaatatctttgattatgattaatgtattatgtcaaaatgtaggctagttaaacttttgtaaagttgcctggaatgtcatttgttaggttataaacacaagatctaaatgaagggttttatgtgttgtgtacaaatcttattttgaaatggacaaacttgtcattacatttgtaaccttgtacagaggatttttcactatgtgcctagcttggtgtccattcagctaaaattgaaaaaaaaaaaaaggtgcatgaagagttaaaaatcaaattaaagtatatgtagagatgactattttatattacatgacccaatcctgtatttatttctaccccctttttgaaagtatttataaaactagttgaggacagctgtatttttttgttgaactatttagtagaattgtgcctttttgtctgtatgtgaataaatgctgtacattttgcaataca

Plasmid map for the 3’UTR construct, Genecopeia


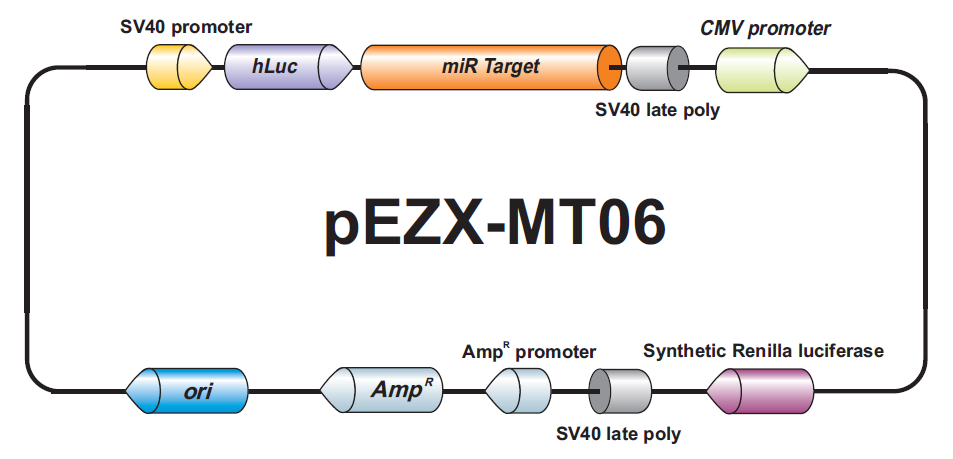

Supplement: Supplementary file 1 [file ijms-24-07825-s001.zip › ijms-2291378-supplementary.docx]
